# Anti-Inflamatory Activity of Neolignan Compound Isolated from the Roots of *Saururus chinensis*

**DOI:** 10.3390/plants9080932

**Published:** 2020-07-23

**Authors:** Sae-Rom Yoo, Hyekyung Ha, Hyeun-Kyoo Shin, Chang-Seob Seo

**Affiliations:** Korea Institute of Oriental Medicine, Daejeon 34054, Korea; k2aori@kiom.re.kr (S.-R.Y.); hkha@kiom.re.kr (H.H.); hkshin@kiom.re.kr (H.-K.S.)

**Keywords:** *Saururus chinensis*, neolignan, inflammation

## Abstract

*Saururus chinensis* (Lour.) Baill. is a perennial herb and grows in Korea, China, and Japan. Interestingly, (7*S*,8*S*)-Δ^8′^-3,4-methylenedioxy-3′,5,5′-trimethoxy-7-monoacetate-8.*O*.4′-neolignan (MTMN), one of the active neolignans, was first isolated from the roots of *Saururus chinensis*. The compound was screened for anti-inflammatory activity using a RAW264.7 murine macrophage cell line. The dried roots of *S. chinensis* (9.7 kg) were extracted with 70% methanol and then solvent fractionation. From the ethyl acetate fraction, MTMN was purified through silica gel column and reverse-phase column chromatography and its structure was identified by spectroscopic analysis with nuclear magnetic resonance, circular dichroism, and mass spectrometry. RAW264.7 cells were induced using lipopolysaccharide (LPS) and treated with or without MTMN. Production of nitric oxide (NO) and prostaglandin E_2_ (PGE_2_) levels were measured and protein expressions of nitric oxide synthase (iNOS) and cyclooxygenase-2 (COX-2) were analyzed by immunoblotting. The isolated neolignan was (7S,8S)-Δ^8′^-3,4-methylenedioxy-3′,5,5′-trimethoxy-7-monoacetate-8.*O*.4′-neolignan. This compound suppressed the LPS-induced iNOS and COX-2 protein expressions, which led to a decrease in the production of NO and PGE_2_ levels. Further studies, including in animal models, will be required to establish the precise pharmacological effect of MTMN.

## 1. Introduction

Inflammation is a biological response that protects the body against harmful stimuli. Inflammatory abnormalities underlie a number of diseases including atherosclerosis [1], mental illness [2,3], sarcopenia [4], and obesity [5]. For over a decade, nonsteroidal anti-inflammatory drugs (NSAIDs) have been commonly used to alleviate inflammatory abnormalities. Although their side effects depend on the specific drug, the adverse effects of long-term use of NSAIDs include an increased risk of gastrointestinal disorders, myocardial infarction, and renal disease [6]. Therefore, recent studies in search of anti-inflammatory agents have focused on plant-derived medications, which are known for having relatively fewer side effects than conventional therapy.

*Saururus chinensis* (Lour.) Baill. (family: Saururaceae), commonly known as “Asian lizard’s tail” [7] is a perennial herb and distributed in parts of East Asia, including Korea, China, and Japan. In Korean folk remedy, it is widely used for the treatment of edema, bladder infection, and several inflammatory diseases [8]. The root of *S. chinensis* contains various kinds of lignans including saucerneol, di-*O*-methyltetrahydrofuriguaiacin B, machilin D, and sauchinone [9,10,11,12], some of which have anti-inflammatory or antioxidant effects [10,13,14]. 

In this study, we isolated a new 8-*O*-4′ type neolignan, (7*S*,8*S*)-Δ^8′^-3,4-methylenedioxy-3′,5,5′-trimethoxy-7-monoacetate-8.*O*.4′-neolignan (MTMN), from the roots of *S. chinensis*. This article presents the isolation, structural elucidation, and anti-inflammatory effects of this compound.

## 2. Results and Discussion

### 2.1. Structure Determination of MTMN

**Figure 1:**
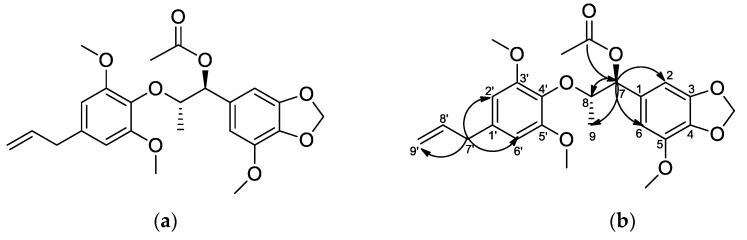
(**a**) Chemical structure and (**b**) key heteronuclear multiple bond correlation of compound **1**.

Compound **1** was obtained as a sticky solid, with a molecular formula of C_24_H_28_O_8_ determined by high-resolution electrospray ionization mass spectrometry (HRESIMS, m/z; found 447.1683 [M+Na]^+^; calcd 447.1682) (Figure S1). Seven methine groups, three methylene groups, two methyl groups, nine quaternary carbons, and three methoxyl groups were identified in the ^1^H-NMR, ^13^C-NMR, DEPT135, and DEPT90 spectrum of 1 (Figure S2). The ^1^H-NMR spectrum exhibited two distinct methyl groups (H-9 and OAc-CH_3_), three methoxyl groups (5-CH_3_, 3’-CH_3_, and 5´-CH_3_), one methylenedioxy group (OCH_2_O-3,4), one methylene group (H-9´), and four aromatic protons (H-2, H-6, H-2´, and H-6´). In the heteronuclear multiple bond correlation spectrum of **1** (Figure 1b and Figure S3), long-range correlations of C-7 with H-2, H-6, H-8 and H-9 were observed. C-7´ showed long-range correlations with H-9´, H-2´, and H-6´. In addition, the ketone group of 7-monoacetate showed a long-range correlation with H-7. The circular dichroism (CD) spectrum (Figure S4) was measured to determine the configurations at C-7 and C-8 of compound **1**. The results revealed that the CD spectrum of **1** showed positive signs in the region of 240–400 nm, indicating the 8S-configuration compared with those of reported analogs [15,16,17]. Furthermore, we confirmed the threo-configuration between H-7 and H-8 by confirming the large coupling constant (*J*_7,8_ = 7.3 Hz) according to previous studies [18,19]. The basis of these data and reported literature, **1**, was determined to be (7S,8S)-Δ^8′^-3,4-methylenedioxy-3′,5,5′-trimethoxy-7-monoacetate-8.*O*.4′-neolignan, which was first isolated and reported in this study.

### 2.2. Inhibitory Effect of Inflammation Related Mediator of MTMN 

Macrophages are a major component of the innate immune system, which modulates inflammatory responses and tissue homeostasis [20]. Lipopolysaccharide (LPS) is a well-known activator of macrophage and activated macrophage leads to the production of inflammatory mediators including cytokines, NO, and prostaglandins [20,21,22]. In this study, we determined the anti-inflammatory mediators affected by MTMN in the LPS-stimulated mouse murine macrophage RAW264.7 cell line. We evaluated the cytotoxicity of MTMN following the treatment of RAW264.7 cells with various concentration for 24 h. MTMN has no toxicity up to 25 μM treatment (data not shown). Thus, nontoxic concentrations of MTMN were used for all subsequent experiments. 

**Figure 2:**
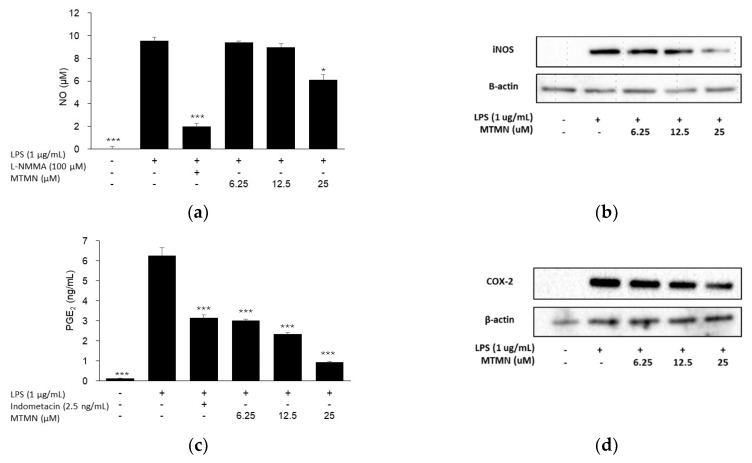
Effect of (7*S*,8*S*)-Δ^8′^-3,4-methylenedioxy-3′,5,5′-trimethoxy-7-monoacetate-8.*O*.4′-neolignan (MTMN) on the production of nitric oxide (NO) (**a**) and prostaglandin E_2_ (PGE2) (**c**), the expression of nitric oxide synthase (iNOS) (**b**) and cyclooxygenase-2 (COX-2) (**d**) in lipopolysaccharide (LPS)-stimulated RAW264.7 murine macrophage cell line. Data are presented as the mean ± SEM. * *p* < 0.05 and *** *p* < 0.001 are compared with the LPS only treated group.

Nitric oxide synthase (iNOS) is produced in response to bacterial products and inflammatory cytokines [21], and iNOS-derived NO is closely related to inflammatory processes. NO plays a role in various physiological and pathological processes, excessive production of NO may lead to inflammation response. As expected, LPS significantly increased the level of NO (9.53 ± 0.34 μM) and the expression of iNOS in RAW264.7 cells (Figure 2a,b). By contrast, MTMN downregulated the expression of iNOS at a concentration of 25 μM. The LPS-stimulated NO level was decreased by MTMN at a concentration of 25 μM by 6.11 ± 0.43 μM (*p* < 0.05). L-NG-monomethyl arginine (L-NMMA), as a non-selective inhibitor of NOS, also significantly decreased the level of NO production by 1.96 ± 0.27 μM in LPS-treated RAW264.7 cell.

Cyclooxygenase (COX) plays important roles in the biosynthesis of PG from arachidonic acid. COX-2 as COX isoform is an early-response gene that is highly inducible by stimuli such as LPS. One of the key downstream products of COX-2, prostaglandin E_2_ (PGE_2_), is a key mediator of inflammatory pain sensitization. Thus, inhibition of COX-2 blocks the synthesis of PGE_2_, and as a result, they suppress inflammation and attenuate pain. As shown in Figure 2c,d, LPS stimulation significantly increased the levels of PGE_2_ (6.26 ± 0.39 ng/mL) and expression of COX-2 compared with the LPS untreated control cell. In contrast, MTMN downregulated the expression of COX-2. Consistent with the immunoblotting results, PGE_2_ secretion was suppressed by MTMN in a dose-dependent manner (3.03 ± 0.05 ng/mL at 6.25 μM, 2.33 ± 0.08 ng/mL at 12.5 μM, and 0.95 ± 0.02 ng/mL at 25 μM). Indomethacin, as an inhibitor of PGE_2_, also decreased the level of PGE_2_ (3.15 ± 0.16 ng/mL) in LPS- treated RAW264.7 cell.

## 3. Materials and Methods 

### 3.1. General Experimental Procedures 

Optical rotation and circular dichroism (CD) were measured using a JASCO DIP-1000 (Tokyo, Japan) automatic digital polarimeter and a JASCO J-1500 spectro-polarimeter (Tokyo, Japan) at 25 °C. The proton nuclear magnetic resonance (^1^H-NMR (250 MHz) and ^13^C-NMR (62.9 MHz)) were used on a Bruker AMX250 spectrometer (Karlsruhe, Germany). Samples were dissolved in CDCl_3_ and reported in ppm downfield from tetramethylsilane. The molecular mass value of the compound was analyzed using Synapt G2 HDMS quadrupole time-of-flight mass spectrometer equipped with an electrospray ion source (Waters, Milford, MA, USA). The stationary phases used for column chromatography (Silica gel 60, 70–230 and 230–400 mesh and Lichroprep RP-18 gel, 40–63 μm, Merck) and thin layer chromatography (TLC) supplemented with 5.5% heat-inactivated fetal bovine serum (Gibco Inc.), penicillin (100 U/mL), and streptomycin (100 μg/mL).

### 3.2. Cytotoxicity Assay 

5 × 10^3^ cells were plated onto a 96-well microplate and treated with various concentrations of MTMN for 24 h. Cytotoxicity of MTMN was detected using a Cell Counting Kit-8 (CCK-8; Dojindo, Kumamoto, Japan). 

### 3.3. Determination of Nitric Oxide (NO) and Prostaglandin E_2_ (PGE_2_) 

RAW264.7 cells were treated with various concentrations of MTMN with lipopolysaccharide (LPS 1 μg/mL) for 18 h. After incubation, the collected supernatant was analyzed for generated nitrite concentration using a Gress reagent system (Promega, Madison, WI, USA). Production of PGE_2_ in the culture supernatant was measured using ELISA kits from Cayman Chemical Co. (Ann Arbor, MI, USA).

### 3.4. Western Blotting

RAW264.7 cells were treated with various concentrations of MTMN, with or without LPS (1 μg/mL), for 18 h. Cells were lysed with a lysis buffer containing protease/phosphatase inhibitor cocktail (Cell Signaling Tech., MA, USA). An equal amount of proteins was resolved by SDS-PAGE gels and then transferred onto polyvinylidene difluoride membranes (Millipore, MA, USA). Inducible nitric oxide synthase (iNOS) and cyclooxygenase (COX)-2 antibodies were used to detect respective proteins and the immunoreactive bands were developed using an enhanced chemiluminescence (ECL) reagent (Thermo Scientific, Rockford, IL, USA). Analyses were carried out in triplicate.

### 3.5. Statistical Analysis 

All experiments were performed in triplicate. The data are presented as mean ± standard error of the mean (SEM). Significant differences were assessed using a one-way analysis of variance and post hoc Bonferroni correction using SYSTAT (Systat Software Inc., Chicago, IL, USA). A *p*-value less than 0.05 (typically ≤ 0.05) is statistically significant.

## 4. Conclusions

The 8.*O*.4′-Type neolignan, (7*S*,8*S*)-Δ^8′^-3,4-methylenedioxy-3′,5,5′-trimethoxy-7-monoacetate-8.*O*.4′-neolignan, was first isolated from the ethyl acetate fraction of the *S. chinensis* root extract. The structure of this compound was determined based on spectroscopic data, NMR, MS, and CD. 

The isolated compound inhibited the LPS-stimulated production of proinflammtory mediators NO and PEG_2_, and related protein expressions of iNOS and COX-2. Further investigation is needed to establish a specific mechanism involved in the anti-inflammatory effect of MTMN. 

## Supplementary Materials

The following are available online at https://www.mdpi.com/2223-7747/9/8/932/s1, Figure S1: HRESIMS spectrum of compound **1**, Figure S2: ^1^H-NMR, ^13^-NMR, DEPT135, and DEPT90 spectra of compound **1**, Figure S3: HMBC spectrum of compound **1**, Figure S4: CD spectrum of compound **1**.
