# Supplementary material for: Anti-Inflamatory Activity of Neolignan Compound Isolated from the Roots of Saururus chinensis
Source: Plants (Basel). 2020 Jul 23;9(8):932. doi: 10.3390/plants9080932 (PMC7466048; doi:10.3390/plants9080932)
Supplement: Supplementary file 1 [file plants-09-00932-s001.pdf]

Supplementary Files

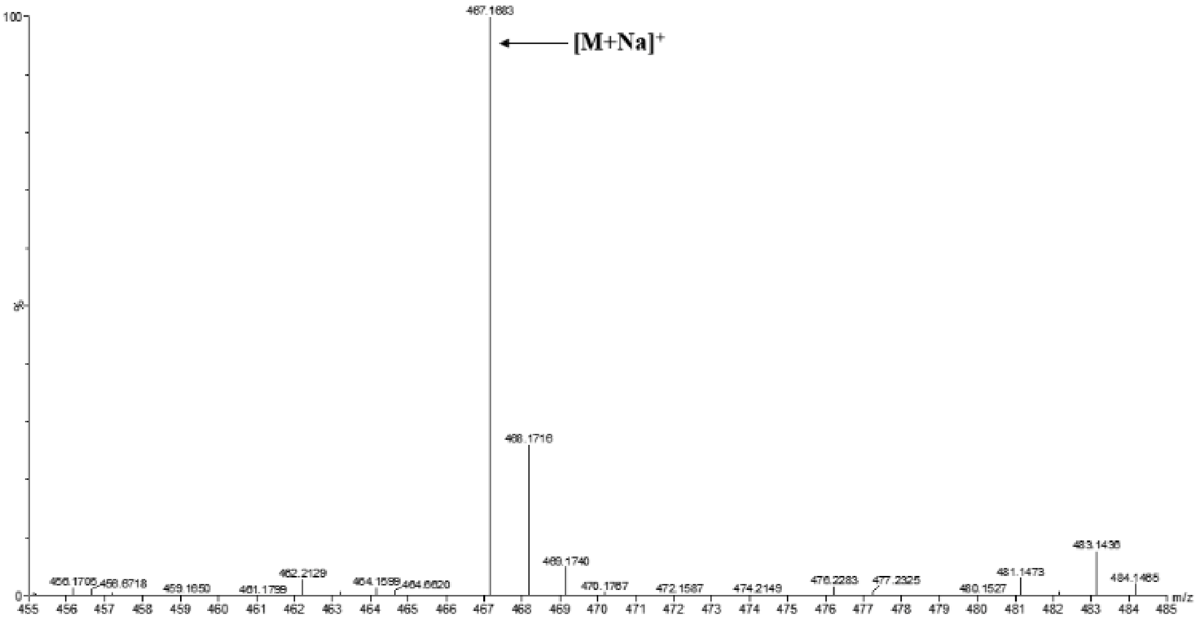

Figure S1. HRESIMS spectrum of compound **1**.

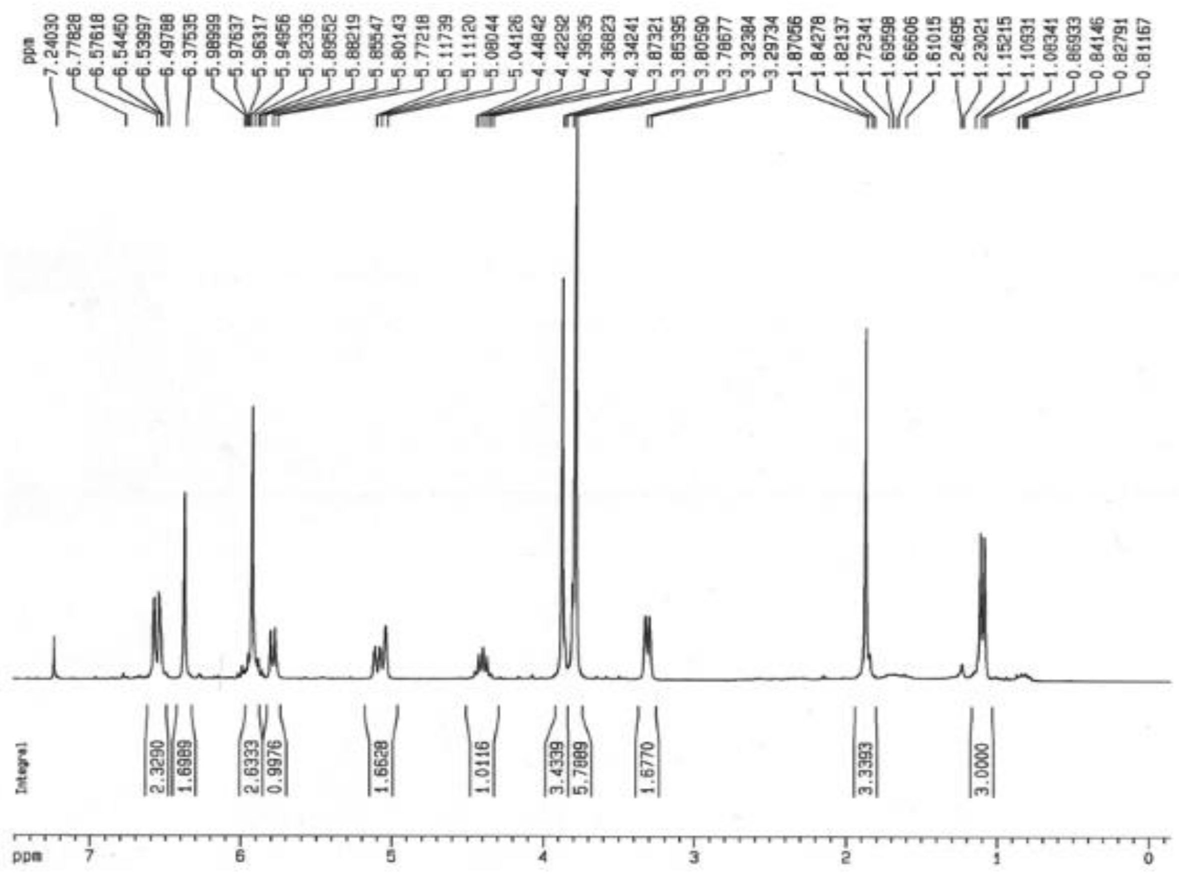
ppm
7.24030
6.77828
6.57618
6.54450
6.53997
6.49788
6.37535
5.98999
5.97637
5.96317
5.94956
5.92336
5.89552
5.88219
5.85547
5.80143
5.77218
5.11739
5.11120
5.08044
5.04126
4.44842
4.42292
4.39635
4.36823
4.34241
3.87321
3.85395
3.80590
3.78677
3.32384
3.29734
1.87056
1.84278
1.82137
1.72341
1.69598
1.66606
1.61015
1.24695
1.23021
1.15215
1.10931
1.08341
0.86933
0.84146
0.82791
0.81167
Integral
2.3290
1.6989
2.6333
0.9976
1.6628
1.0116
3.4339
5.7889
1.6770
3.3393
3.0000
ppm
7
6
5
4
3
2
1
0

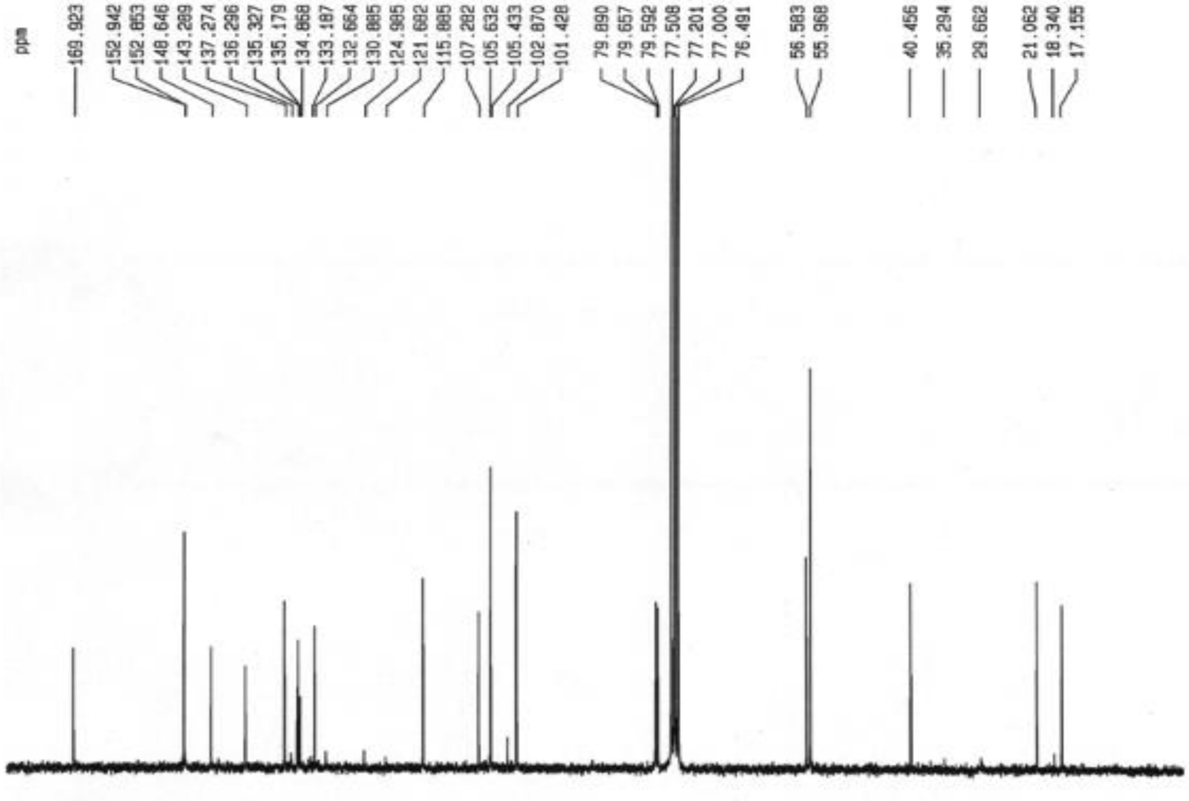
ppm
169.923
152.942
152.853
148.646
143.289
137.274
136.296
135.327
135.179
134.868
133.187
132.664
130.885
124.985
121.682
115.885
107.282
105.632
105.433
102.870
101.428
79.890
79.657
79.592
77.508
77.201
77.000
76.491
56.583
55.968
40.456
35.294
29.662
21.062
18.340
17.155
ppm
160
140
120
100
80
60
40
20

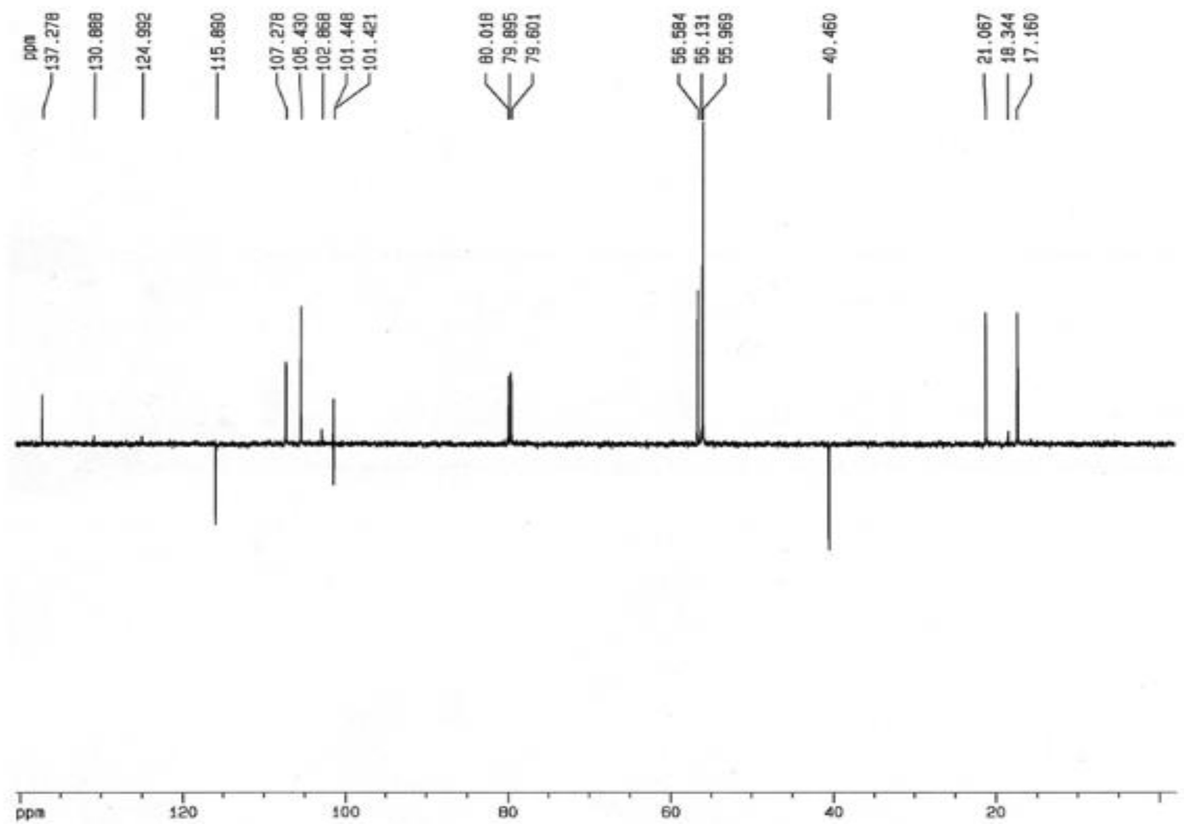

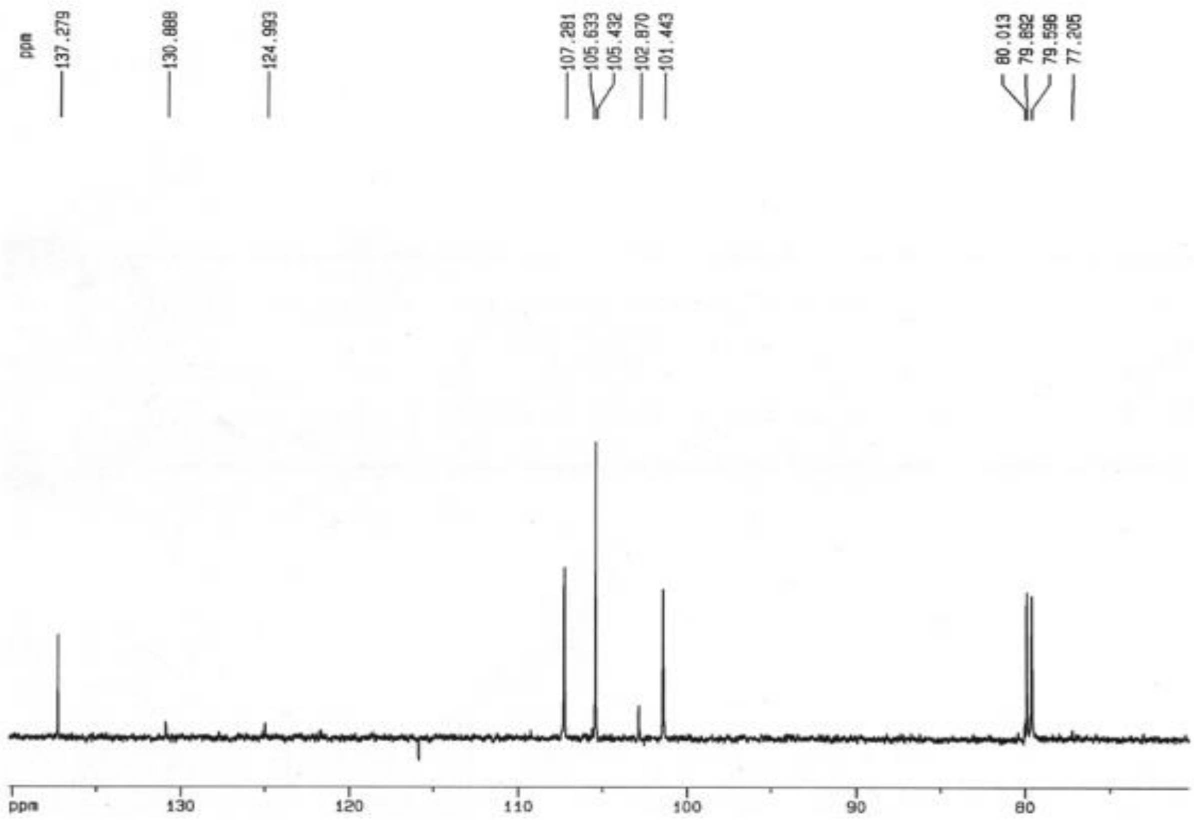

Figure S2. ^{1}H-NMR, ^{13}-NMR, DEPT135, and DEPT90 spectra of compound **1**.

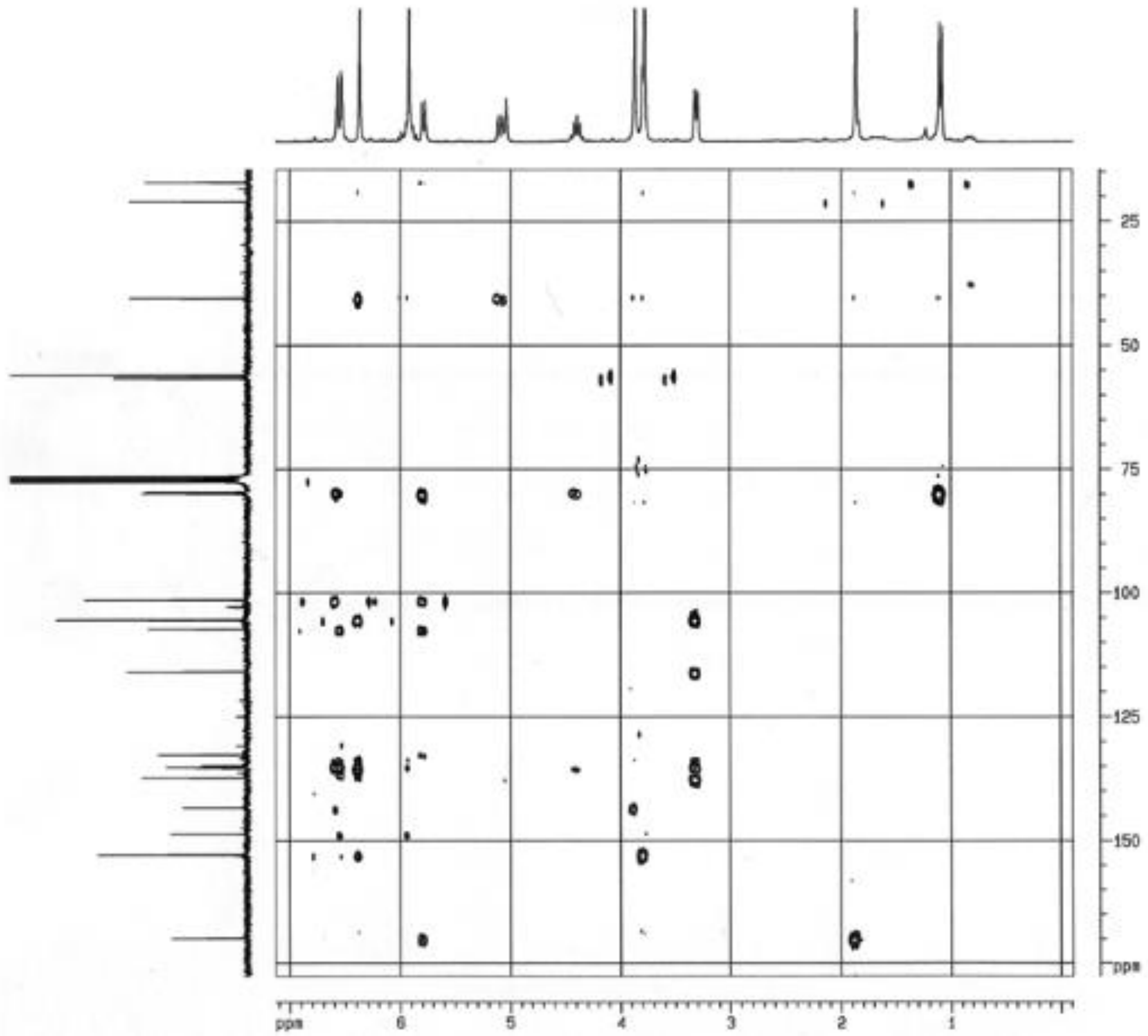

Figure S3. HMBC spectrum compound **1**.

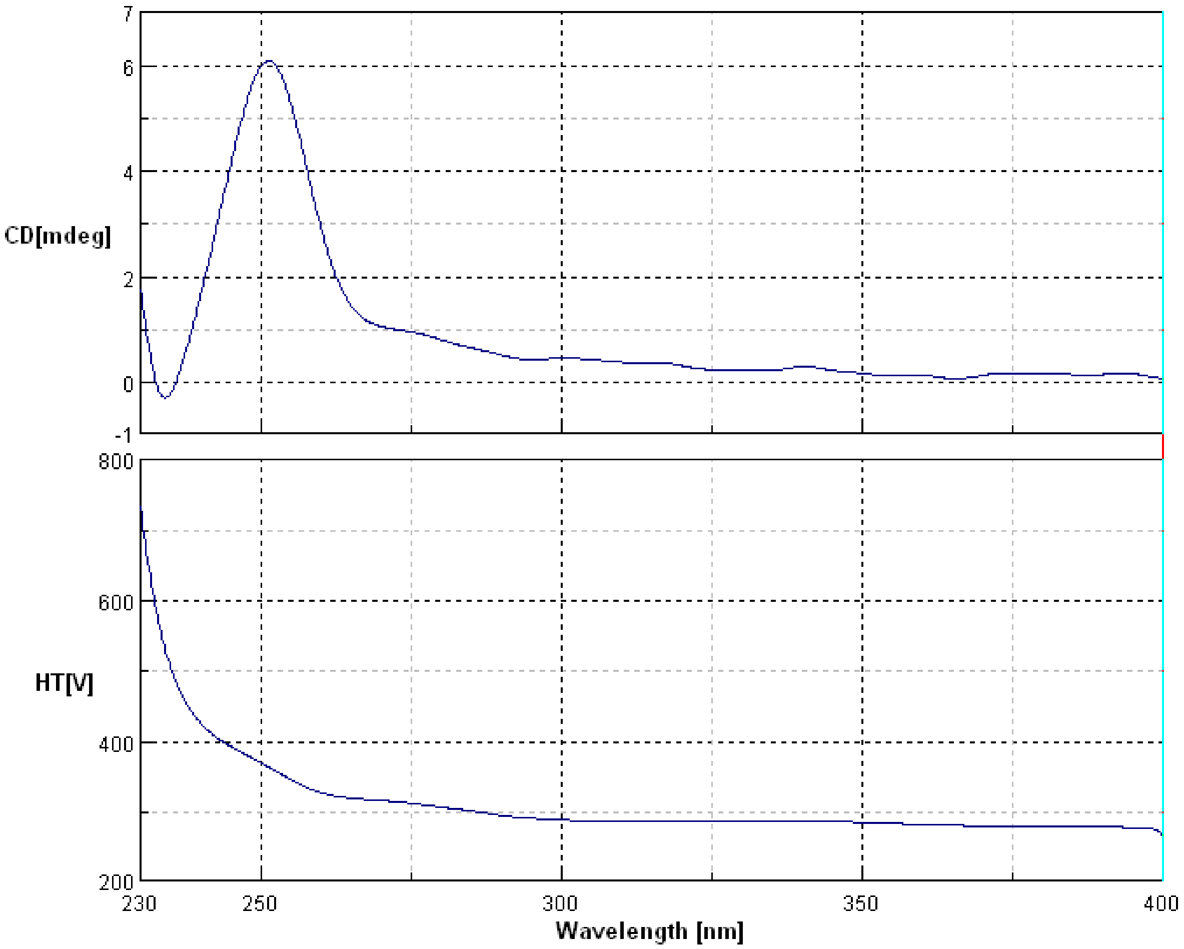

Figure S4. CD spectrum compound **1**.
